# Neutrophil Extracellular Traps are Involved in the Innate Immune Response to Infection with *Leptospira*

**DOI:** 10.1371/journal.pntd.0003927

**Published:** 2015-07-10

**Authors:** Emilia Scharrig, Agostina Carestia, María F. Ferrer, Maia Cédola, Gabriela Pretre, Ricardo Drut, Mathieu Picardeau, Mirta Schattner, Ricardo M. Gómez

**Affiliations:** 1 Laboratory of Animal Viruses, Institute of Biotechnology and Molecular Biology, CCT-La Plata, CONICET-UNLP, Buenos Aires, Argentina; 2 Laboratory of Experimental Thrombosis, Institute of Experimental Medicine, CONICET-National Academy of Medicine, Buenos Aires, Argentina; 3 Division of Pathology, Children Hospital “Superiora Sor María Ludovica”, La Plata, Argentina; 4 Cátedra de Patología “A”, Facultad de Medicina, Universidad Nacional de La Plata, La Plata, Argentina; 5 Institut Pasteur, Biology of Spirochetes Unit, Paris, France; University of California San Diego School of Medicine, UNITED STATES

## Abstract

NETosis is a process by which neutrophils extrude their DNA together with bactericidal proteins that trap and/or kill pathogens. In the present study, we evaluated the ability of *Leptospira* spp. to induce NETosis using human *ex vivo* and murine *in vivo* models. Microscopy and fluorometric studies showed that incubation of human neutrophils with *Leptospira interrogans* serovar Copenhageni strain Fiocruz L1-130 (LIC) resulted in the release of DNA extracellular traps (NETs). The bacteria number, pathogenicity and viability were relevant factors for induction of NETs, but bacteria motility was not. Entrapment of LIC in the NETs resulted in LIC death; however, pathogenic but not saprophytic *Leptospira* sp. exerted nuclease activity and degraded DNA. Mice infected with LIC showed circulating NETs after 2 days post-infection (dpi). Depletion of neutrophils with mAb1A8 significantly reduced the amount of intravascular NETs in LIC-infected mice, increasing bacteremia at 3 dpi. Although there was a low bacterial burden, scarce neutrophils and an absence of inflammation in the early stages of infection in the kidney and liver, at the beginning of the leptospiruric phase, the bacterial burden was significantly higher in kidneys of neutrophil-depleted-mice compared to non-depleted and infected mice. Surprisingly, interstitial nephritis was of similar intensity in both groups of infected mice. Taken together, these data suggest that LIC triggers NETs, and that the intravascular formation of these DNA traps appears to be critical not only to prevent early leptospiral dissemination but also to preclude further bacterial burden.

## Author Summary

Neutrophils extracellular traps (NETs) are a relatively novel pathogen-killing mechanism for extracellular microbes independent of phagocytic uptake and degranulation. Although it was originally proposed that NETs are formed exclusively in tissues at sites of infection, NETs have also been found within blood vessels where they ensnare microbes in circulation during sepsis. Leptospirosis is a global zoonosis that has become prevalent in slum areas, where its diagnosis and treatment may be overlooked. Here, for the first time, we characterized NETs formation as a result of *Leptospira* spp. stimulus. We also showed that mice lacking neutrophils exhibit early reduced levels of circulating NETs, but higher bacteremia, a fact that was later associated with higher bacterial burden in kidney. The present results demonstrate that *Leptospira* spp. can trigger NET formation and that this innate immune mechanism can play a role in the pathogenesis of leptospirosis.

## Introduction

Leptospirosis is an important global zoonosis that is more frequent in tropical and subtropical areas, caused by pathogenic spirochetes of the genus *Leptospira* [1]. Historically, the transmission of leptospirosis was mainly associated with exposure of individuals to wild or farm animals [2,3]. Humans usually become infected through contact with urine-contaminated soil and water or with infected animal tissues [4,5]. However, in recent decades, leptospirosis has become an emerging disease that is prevalent in cities with sanitation deficits and a large population of urban rodent reservoirs [6,7]. Therefore, leptospirosis has become a neglected tropical disease with an endemo-epidemic pattern associated with slum settlements where deficiencies in the sanitation infrastructure are maximal [8,9].

The pathogenesis of leptospirosis is not completely understood. It is accepted that pathogenic *Leptospira* spp. have the ability to rapidly disseminate via the blood to many organs of the host during the leptospiremic phase of infection [10]. If the host survives, the leptospiruric phase begins 5–10 days later with the increase of specific circulating antibodies after 5–7 days, which clear the bacteria in blood and most organs except the kidney, where bacteria can persist in the proximal tubules for months. As a result, an interstitial nephritis may develop, affecting kidney physiology to varied degrees, and the host will become a carrier that contaminates the environment with their urine.

The extracellular release of the nuclear DNA of neutrophils upon activation by bacterial or fungal species is a relatively novel pathogen-killing mechanism of neutrophils [11,12]. These DNA structures, named neutrophil extracellular traps (NETs), are composed of chromatin, associated with several proteins with antimicrobial properties such as histones, elastase and myeloperoxidase, among others, and serve as a physical barrier that prevents the further spread of pathogens [13]. Thus, NETs immobilize and even kill extracellular microbes independently of phagocytic uptake and degranulation. Although it was originally proposed that NETs are formed exclusively in tissues at sites of bacterial or fungal infection, NETs have also been found within blood vessels (specifically in the lung capillaries and liver sinusoids) where they ensnare bacteria in circulation during sepsis [14]. The role of NETs in the pathogenesis of human leptospirosis is unknown.

Here, we studied and partially characterized *Leptospira* spp. as a stimulus for NET formation using human *ex vivo* and murine *in vivo* models. Our results suggest that NETs formation is an important mechanism in the pathogenesis of leptospirosis.

## Materials and Methods

### Bacteria

The virulent *Leptospira interrogans* serovar Copenhageni (LIC) strain Fiocruz L1–130 [9], *Leptospira interrogans* serovar Manilae (LIM) strain L495 and their mutant *fla*A2 [15], the avirulent *Leptospira biflexa* serovar Patoc strain Patoc 1 [16], and their mutant *fla*B [17] have been described previously. The bacteria were cultured at 30°C under aerobic conditions in liquid Ellinghausen-McCullough-Johnson-Harris (EMJH) medium (Difco, USA), supplemented with 10% rabbit serum (vol/vol), 0.015% L-asparagine (wt/vol), 0.001% sodium pyruvate (wt/vol), 0.001% calcium chloride (wt/vol), 0.001% magnesium chloride (wt/vol), 0.03% peptone (wt/vol) and 0.02% meat extract (wt/vol) [16]. The bacteria were quantified by direct counting in a Petroff-Hausser chamber (Hausser Scientific, UK) using dark field microscopy. For inactivation studies, bacteria were centrifuged at 20,000 xg for 10 min, resuspended in 4% PFA for 10 min followed by two washes with PBS or were inactivated by heat at 80°C for 20 min, followed by centrifugation and washing.

### Reagents

Phorbol 12-myristate 13-acetate (PMA) and propidium iodide (PI) were purchased from Sigma Aldrich (St. Louis, MO). Cellstar culture microplates were purchased at Greiner Bio-One (Monroe, NC). Micrococcal nuclease (MNase) and the cell death detection enzyme-linked immunosorbent assay (ELISA) plus kit were obtained from Roche Diagnostics (Mannheim, Germany). Recombinant histone H4 (rH4) was obtained from Biolabs (UK, cat# M2504S). The recombinant protein was produced in a transformed *E*. *coli* strain carrying an expression vector encoding the human histone H4 gene (HIST2H4). The concentration was 1mg/ml (89 μM). Rat mAb (1A8) anti-Ly6G was obtained from BioXCell (NH, USA). Murine polyclonal anti-LipL32 antiserum was generously provided by Dr. ALTO Nascimento, Instituto Butantan, SP, Brazil. Rabbit anti-neutrophil elastase was obtained from Calbiochem-Merk Millipore (Darmstad, Germany), anti-mouse Alexa 488 and anti-rabbit Alexa 488 was obtained from Invitrogen Molecular Probes (Eugene, OR, USA). Biotinylated goat anti-mouse or rat IgG was obtained from Dako (Glostrup, Denmark). Peroxide-labeled streptavidin was obtained from Dako (CA, USA). DNase I was purchased from Biodynamics (Bs As, Argentina).

### Isolation of human neutrophils

This study was conducted according to the principles expressed in the Declaration of Helsinki. All healthy patients provided written informed consent for the collection of samples and subsequent analysis. Neutrophils were isolated from peripheral blood drawn from healthy donors by Ficoll-Hypaque gradient centrifugation and dextran sedimentation, as described previously [18]. Cell suspensions contained more than 96% neutrophils, as determined by May Grünwald-Giemsa stained cytopreps, and the levels of monocyte contamination were always 0.2%, as evaluated by CD14 staining and flow cytometry. The cells (5x10^5^/mL) were resuspended in RPMI 1640 medium supplemented with bovine serum albumin (2%).

### NET formation assay

NET formation assays have been performed as previously described with minor modifications [18]. Briefly, neutrophils (2x10^5^) were seeded in 24-well flat-bottom-plates with poly-L-lysine-treated coverslips, at pH 7.4, and placed in a humidified incubator at 37°C with CO_2_ (5%). After stimulation with PMA or *Leptospira* spp for 180 minutes, NET formation was visualized by immunofluorescence microscopy and quantified by evaluation of the DNA released in the supernatants by fluorometry or by measuring nucleosomes with an ELISA kit (Roche).

### Immunochemical staining

After PMA or *Leptospira* spp. stimulation, cells were fixed with 4% paraformaldehyde (PF), permeabilized with Triton x-100 (0.25%), stained with PI (2 mg/mL) washed and mounted on slides with polymount (Polysciences, PA, USA). In selected experiments, the generation of NETs was confirmed by labeling the cells with PI and an antibody against elastase. Images for NET evaluation were taken and analyzed by fluorescence microscopy, using a Nikon E200 photomicroscope. Immunohistochemistry was performed as previously described [19]. Briefly, the slides were incubated for 1 h at 37°C with the respective primary antiserum diluted 1:200. After several washes with PBS, samples were incubated with a biotinylated secondary anti-species IgG antibody. Samples were washed and then incubated with peroxide-labeled streptavidin. Incubation with diaminobenzidine/hydrogen peroxidase substrate (DAB) was allowed to reach the appropriate intensity and slides were counterstained with hematoxylin, mounted and observed on a Nikon E200 photomicroscope.

### Quantification of extracellular DNA

DNA released from neutrophils during NETosis was digested with MNase (500 mU/ml) for 15 min in the presence of CaCl_2_ (1.5mM). EDTA (5 mM) was added to stop the nuclease activity. Supernatants were collected, centrifuged and DNA was measured in the supernatants using sybr gold in a fluorometer (Biotek, Winooski, VT, USA). The calibration curve was constructed using thymus DNA of known concentration. The obtained data was confirmed using an ELISA kit for nucleosomes (Roche).

### Histopathology

Samples were processed for routine histology and kidney injury was scored as previously described [19]. Briefly, nephritis was graded blindly by a pathologist on a scale of 0–4 in a whole longitudinal section of the organ: a score of 0 corresponded to an absence of inflammatory infiltrates or necrosis, 1 denoted minimal inflammation (1 to 5 foci), 2 indicated mild inflammation (< 25% of the section affected or 6–10 foci), 3 suggested moderate inflammation (25–50% of the section affected or 11–20 foci), and 4 corresponded to severe inflammation (more than 50% of the section with inflammatory infiltrates or more than 20 foci).

### DNA isolation

Kidney samples were subject to mechanical homogenization in 500 μl of lysis buffer (50 mM Tris-HCl pH 8.0; 1 μM EDTA, 1% Triton X-100, 0.5% Tween-20; 1% SDS) containing proteinase K (2 μg/ml) and incubated for 2 h at 56°C. One volume of phenol (pH 8.0) was added and centrifuged at 12,000 xg for 15 min at 4°C. The aqueous phase was extracted once more with phenol and then four times with 0.5 volumes of chloroform/isoamyl alcohol (24:1), with centrifugation at 12,000 xg for 10 min at 4°C between each step. DNA was precipitated overnight at -20°C by the addition of 100 μl of 3 M sodium acetate pH 5.2 and 100% ethanol up to a volume of 1 mL, and then centrifuged at 12,000 xg for 20 min at 4°C. The DNA pellet was washed twice with 70% ethanol, resuspended in TE buffer (10 mM Tris-HCl pH 7.5, 1 mM EDTA) and incubated at 55–60°C for 15–20 min. DNA was stored at -20°C until use [19].

### Real time-PCR

The real time-PCR (qPCR) studies were performed with a Line-Gene K instrument and software (Bioer, China). The 5x hot firepol Eva green qPCR mix plus (Solis BioDyne, Estonia) was used for all reactions, following the manufacturer’s instructions. Initial denaturation was carried out at 94°C for 10 min, followed by 65 cycles of 20 s at 94°C, 15 s at the respective annealing temperatures and 15 s at 72°C each, and a final extension at 72°C for 2 min. A melting curve analysis was performed immediately after amplification at a linear temperature transition rate of 0.3°C/s from 70°C to 89°C with continuous fluorescence acquisition. The size of PCR products was confirmed by agarose gel electrophoresis. To obtain bacterial burden values, 16SDNA bacterial gene was amplified and the number of bacteria was referred to that of the host cells [19]. The primer sequences were (5’>3’) Fw: CATTCATGTTTCGAATCATTTCAAA, and Rv: GAAACACGGACACCCAAAGTA; the size of the amplified fragments was 331 bp.

### Animals

Ethics Statement: all animal experiments were in compliance with the Argentine animal protection Law “*Ley 14346 –Malos tratos y actos de crueldad a los animales*”. The ethics committee of the “Instituto de Biotecnología y Biología Molecular, CONICET-UNLP”, in agreement with the International Guiding Principles for Biomedical Research Involving Animals (NIH, 1985), did not raise any concerns and approved our research protocol (identification number 001/12). All animals received water and food *ad libitum*. All efforts were made to minimize suffering.

Four-week-old C57BL/6J inbred male mice were purchased from the Faculty of Veterinary, National University of La Plata, Argentina.

### Neutrophil depletion assays

For the depletion of Ly6G^high^ neutrophils *in vivo*, C57BL/6J mice were treated with purified anti-Ly6G rat mAb (1A8, BioXCell, NH, USA). Intraperitoneal (i.p.) injections (0.5 mg/200 μl PBS) were administered to achieve 90–95% depletion of circulatory neutrophils, as previously described by others [20]. Mice were treated 24 h prior to infection and every 48 h thereafter. Control animals received a similar dose of purified whole rat IgG (Jackson Laboratories, MA, USA). Granulocytes were quantified with a veterinarian hematology counter every 48 h.

### Quantification of circulating NETs

For the quantification of circulating nucleosomes, plasma samples from infected mice were collected at different times post-infection. Nucleosomal DNA or complexes of DNA bound to histones were measured using an ELISA kit according to the manufacturer’s instructions. The calibration curve was constructed using a standard of nucleosomal DNA of known concentration.

### Viability assays

LIC was incubated alone (1x10^7^), with neutrophils (2x10^5^/mL, MOI 50), or with neutrophils and DNase I (0,25U/mL), for 180 min. Then, bacterial viability was quantified by the MTT colorimetric method.

For the rH4 experiments, LIC (MOI 50) was incubated with 0, 0.5, 1, 2 or 4 μM of rH4 for 60 min at room temperature in PBS, and then bacterial viability was quantified with the Live/dead Baclight bacterial viability and counting kit (Life Technology, Thermo Fisher Scientific, USA), following the manufacturer’s instructions and expressed as a percentage of the 0 value.

The colorimetric 3-(4,5-dimethylthiazol-2-yl)-2,5-diphenyl tetrazolium bromide (MTT) assay was performed to test the survival of the *Leptospira* spp. as described previously [21] with minor changes. Briefly, after incubation with neutrophils, cells were collected in EMJH and incubated for 4 days at 30°C. Then, the bacteria were washed and 50 μL of MTT (5 mg/mL) were added and incubated for 45 min at 37°C. The formazan crystals formed in the living cells were dissolved in 200 μL of DMSO. Absorbance of the solution was measured at 510 nm using a microplate reader (TP-reader, Thermo).

### DNA degradation studies

Aliquots (50 ng) of an already described plasmid DNA of approximately 11 Kb [22] were incubated with PBS (negative control), or 0,25U of DNase I (positive control), or 1x10^7^ *L*. *interrogans* serovar Copenhageni (LIC) or same number of *L*. *biflexa* serovar Patoc (Patoc) at 37°C for 60 min. After incubation, the samples were boiled with sample buffer (0.25% bromophenol blue, 40% w/v sacarose) and loaded onto a 0.8% agarose gel and run in TAE buffer (40 mM Tris, 20 mM acetic acid, 1mM EDTA) with 1% ethidium bromide at 90 volts. The electrophoretical migration of the bands was observed in an UV light transilluminator (Fotodyne o Stratagen, USA), and images were captured with a Kodak DC120 camera and the Electrophoresis Documentation and Analysis System 120 software (Kodak DC120).

### Statistical analysis

All results are expressed as the mean plus SEM. Student’s paired t test was used to determine the significance of differences between means, and p values lower than 0.05 were considered to be statistically significant. When multiple groups were compared, two and one-way analysis of variance (ANOVA) followed by the Bonferroni procedure was used to determine significant differences between groups. All statistical analyses were performed by using Prism 6 software (GraphPad).

## Results

### *Leptospira interrogans* induce NETs in a concentration-dependent manner

**Fig 1:**
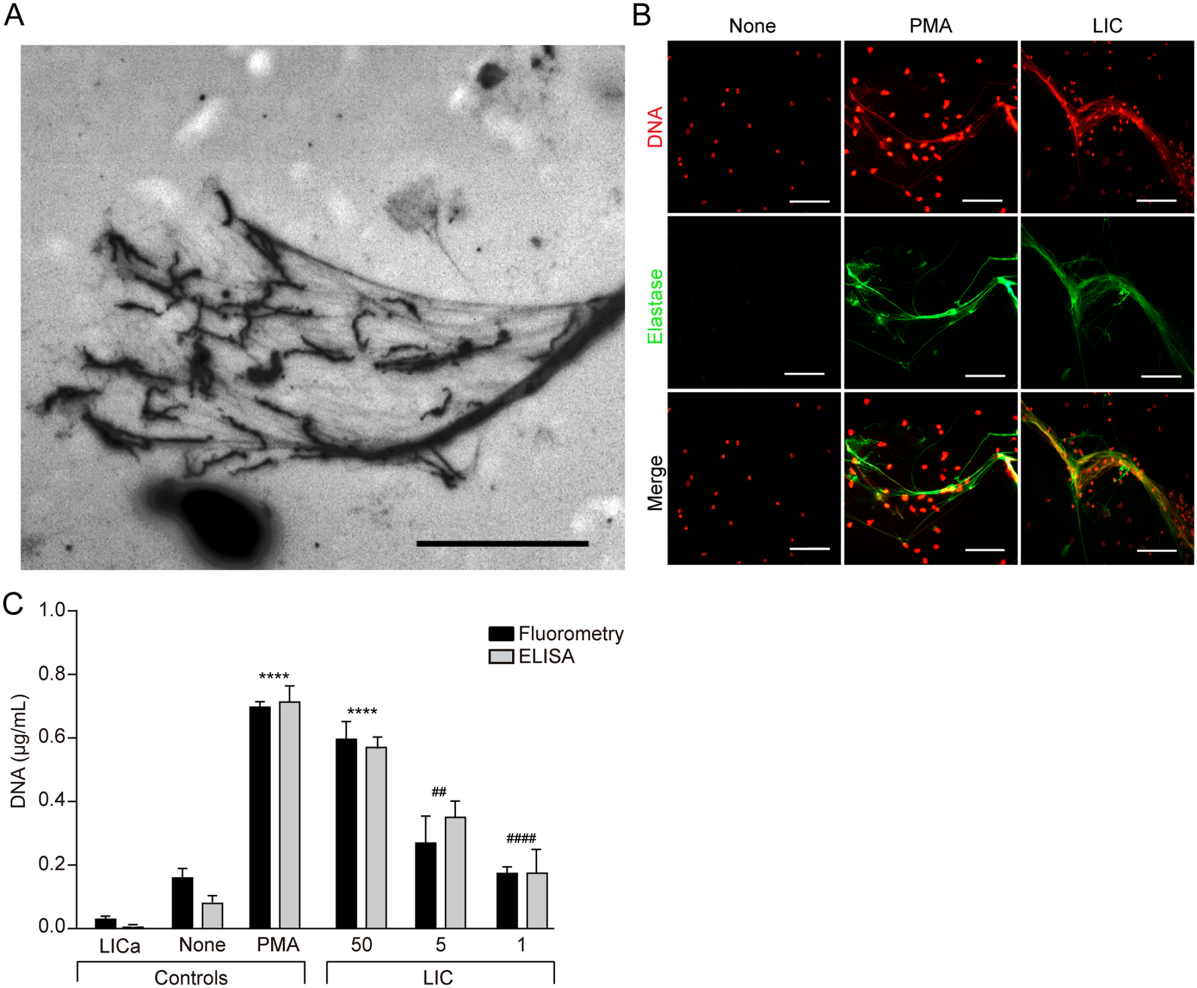
*Leptospira interrogans* induce NETs in a concentration-dependent manner. Human neutrophils (2x10^5^/mL) were incubated with *Leptospira interrogans* serovar Copenhageni (LIC) (MOI = 50) for 180 min and (A) visualized with a Nikon E200 photomicroscope or (B) fixed (PF 4%) and stained with propidium iodide (red) or with the specific marker anti-neutrophil elastase (green), and analyzed by fluorescence microscopy (n = 10). Scale bar indicates 50 μm. (C) DNA or nucleosomes were measured by fluorometry (black bars) or ELISA kit (white bars) respectively in supernatants of LICa (bacteria alone) or unstimulated neutrophils (None) (500/μL) (negative control), stimulated with PMA (50 ng/mL) (positive control), or with LIC (MOI 50, 5 or 1) for 180 min Bars represent standard error of the mean (SEM) of assays from 3–10 independent assays; ***p <0.0001 vs. None. ##p<0.01 and ####p<0.0001 vs. LIC 50.

In order to investigate whether *Leptospira* sp. was able to induce NETs, we performed a series of initial experiments using *Leptospira interrogans* serovar Copenhageni (LIC) and human neutrophils. Stimulation of neutrophils with LIC resulted in the release of fine strands of extracellular DNA with bacteria trapped at their ends (Fig 1A). Using PMA as a positive control, the generation of NETs was confirmed by double-labeling studies with PI, to identify DNA, and with an antibody against elastase, a major neutrophil granular-derived protein that decorates the DNA strands (Fig 1B). Quantitative assays of the released DNA or nucleosomes indicated that the formation of NETs was dependent on bacterial concentration (Fig 1C). DNA levels in supernatants of LIC alone (without neutrophils) were not significant (Fig 1C).

### Pathogenicity and viability, but not motility, of *Leptospira* spp. are relevant factors for NET formation

**Fig 2:**
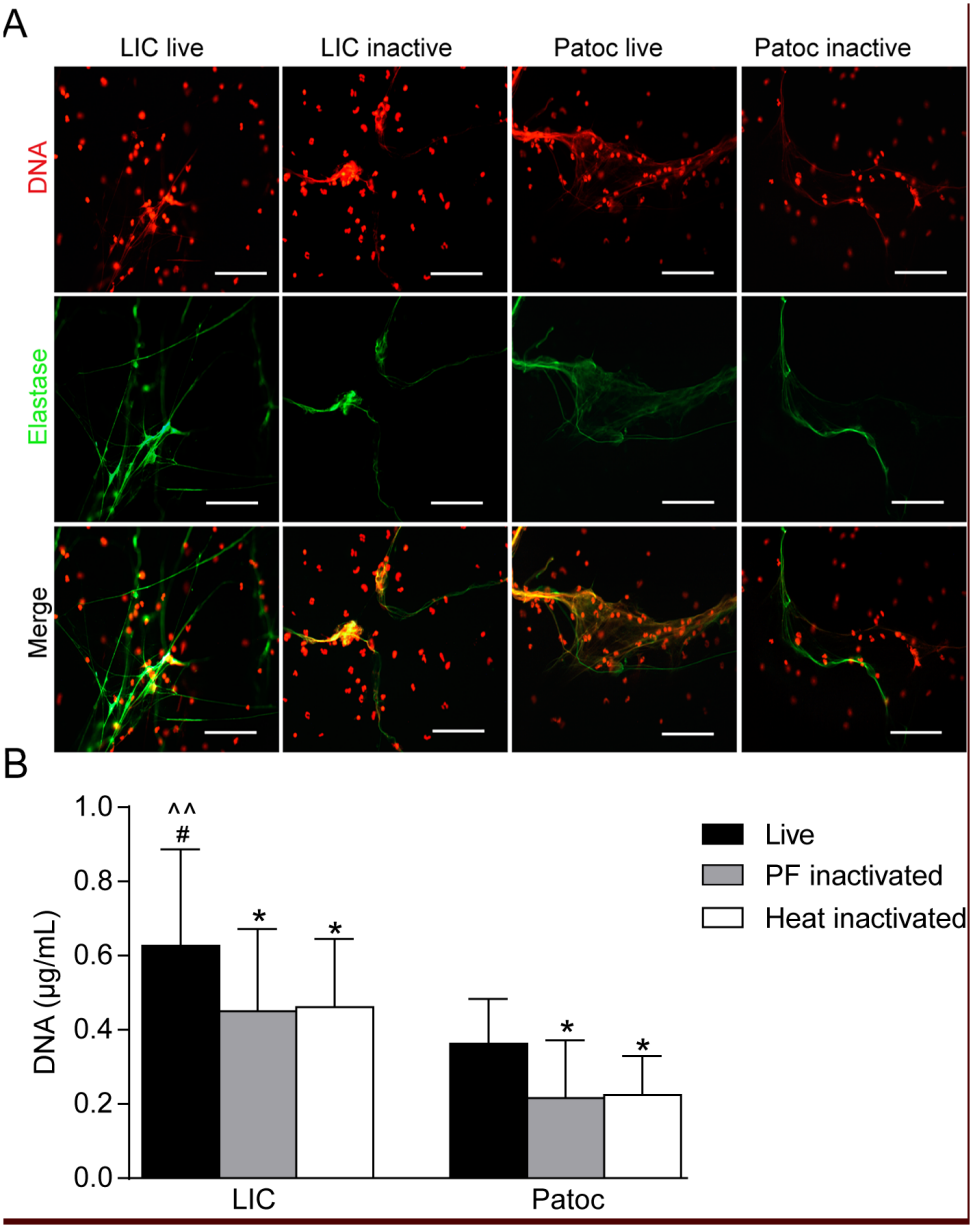
Pathogenicity and viability are relevant factors for NET formation. **(A)** Human neutrophils (2x10^5^/mL) were incubated with live or inactivated with 4% PF or by heat *Leptospira interrogans* serovar Copenhageni (LIC) or *Leptospira biflexa* serovar Patoc (Patoc) (MOI = 50) for 180 min and then fixed (PF 4%), stained with propidium iodide (red) or with the specific marker anti-neutrophil elastase (green), and analyzed by fluorescence microscopy (n = 10). Scale bar indicates 50 μm. **(B)** Quantification of NETs released by fluorometry in the same conditions as in **(A)**. Bars represent standard error of the mean (SEM) of assays from ten independent assays; *p <0.05, ** *p* <0.01.

To test whether pathogenicity and viability modulated NET formation, neutrophils were exposed to the pathogenic LIC and the saprophytic *Leptospira biflexa* serovar Patoc (Patoc) either alive or after inactivation with 4% PF for 10 min. Although both *Leptospira* spp. triggered NETs, significantly higher levels were induced by pathogenic and live *Leptospira* (Fig 2A and 2B). Similar values were obtained with heat-inactivated bacteria (Fig 2B).

**Fig 3:**
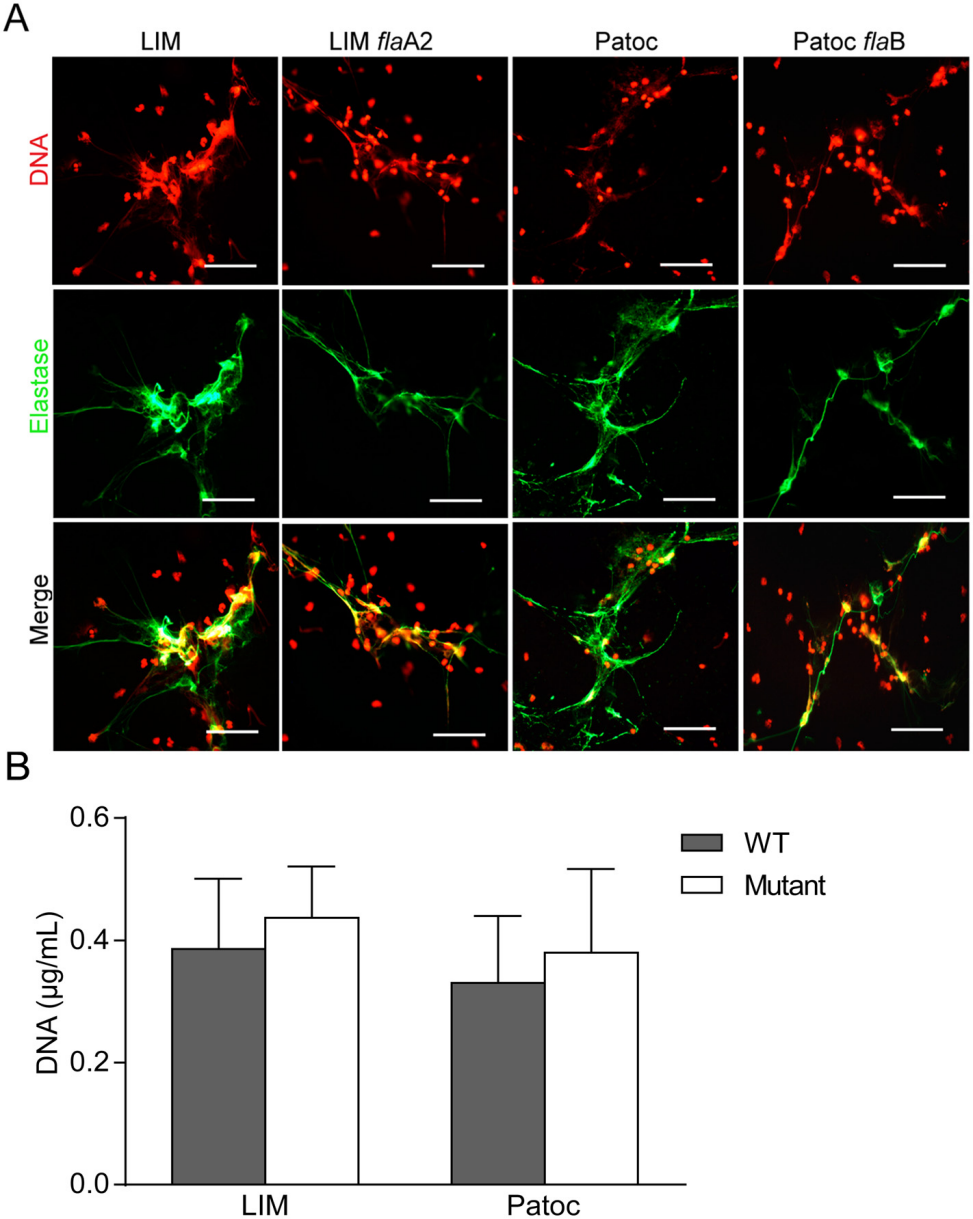
*Leptospira* spp. motility is not a relevant factor for NET formation. **(A)** Human neutrophils (2x10^5^/mL) were incubated with *Leptospira interrogans* serovar Manilae (LIM) and *Leptospira biflexa* serovar Patoc (Patoc) and their non-mobile mutants *fla*A2 and *fla*B (MOI = 50) for 180 min and then fixed (PF 4%) and stained with propidium iodide (red) or with the specific marker anti-neutrophil elastase (green) and analyzed by fluorescence microscopy (n = 10). Scale bar indicates 50 μm. **(B)** Quantification of NETs released by fluorometry in the same conditions as in **(A)**.

Considering that *Leptospira* spp. are highly motile bacteria, we explored whether this property was important for the induction of NETs. In order to achieve this aim, *Leptospira interrogans* serovar Manilae (LIM) *fla*A2 and Patoc *fla*B mutants (non-motile) were compared with their motile parental strains. As shown in Fig 3A and 3B, bacteria motility does not appear to be relevant for NET formation since there were no significant differences in the formation of NETs triggered by the wild-type or the mutant strains.

### NETs kill *Leptospira interrogans*

**Fig 4:**
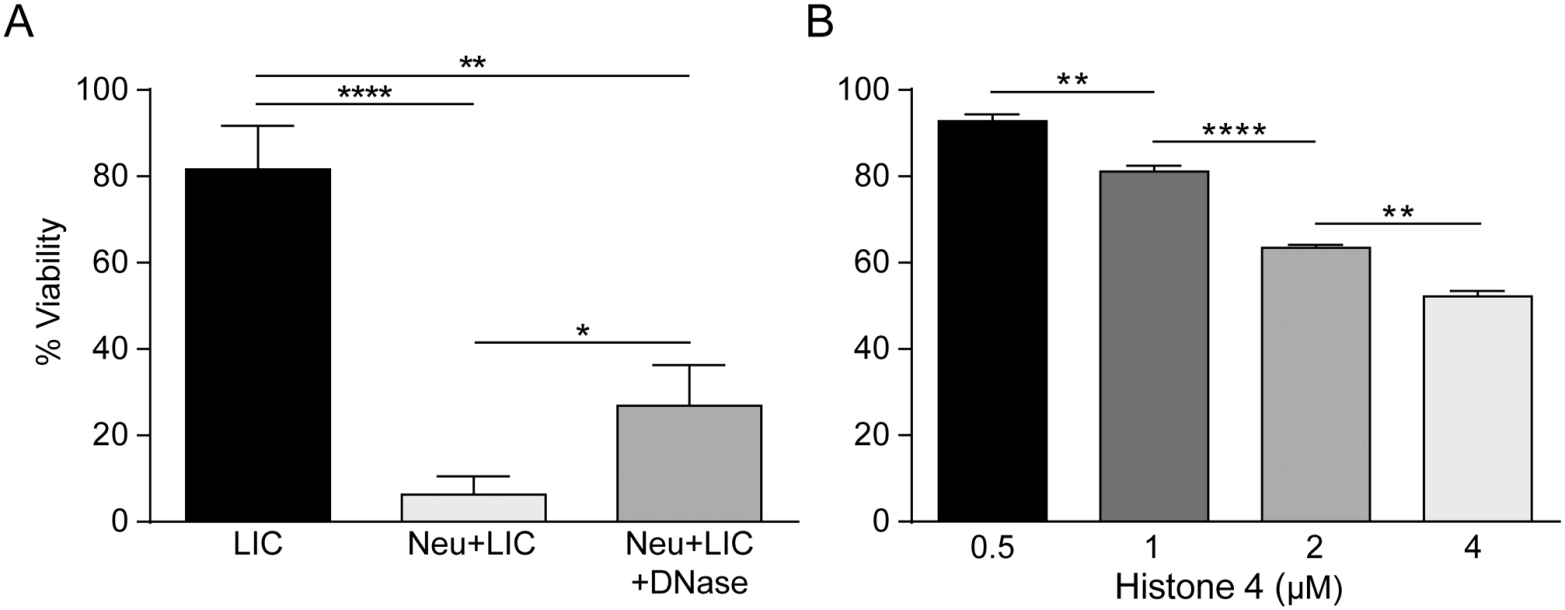
NETs kill *Leptospira* sp. **(A)** Percentage viability after 180 minutes of *Leptospira interrogans* serovar Copenhageni (LIC) (MOI = 50) alone, after being incubated with human neutrophils (Neu) (2x10^5^/mL), or in the presence of DNase (0.25 U/mL). **(B)** Percentage viability of LIC (MOI = 50) after 60 minutes of incubation with different concentrations of recombinant histone H4. Bars represent standard error of the mean (SEM) of assays from five independent assays; *p <0.05, **p <0.01, ****p <0.0001.

Considering the fact that it has been reported that NETs might only trap [23] or trap and kill bacteria [11], we performed a set of experiments where neutrophils were incubated with LIC followed by analyses of bacteria viability after NET formation. Results showed that NETs reduced the viability of LIC by more than 90%. The degradation of LIC-induced NETs using DNase treatment at the beginning of the assay increased the number of live bacteria (Fig 4A).

Since the release of LIC from DNA traps by DNase treatment did not result in the full recovery of LIC viability, and it is known that, among several proteins present in the NET, histones exert bactericide activity [24], we next examined the ability of histones to kill *Leptospira* sp. To address this point, a fixed number of LIC were incubated with various concentrations of recombinant histone H4, which has been shown to be the most potent in triggering cellular responses [25,26], and bacterial viability was determined 1 h later. Fig 4B shows that histone H4 induced bacterial death in a concentration-dependent manner.

### Pathogenic but not saprophyte *Leptospira* spp. degrades DNA

**Fig 5:**
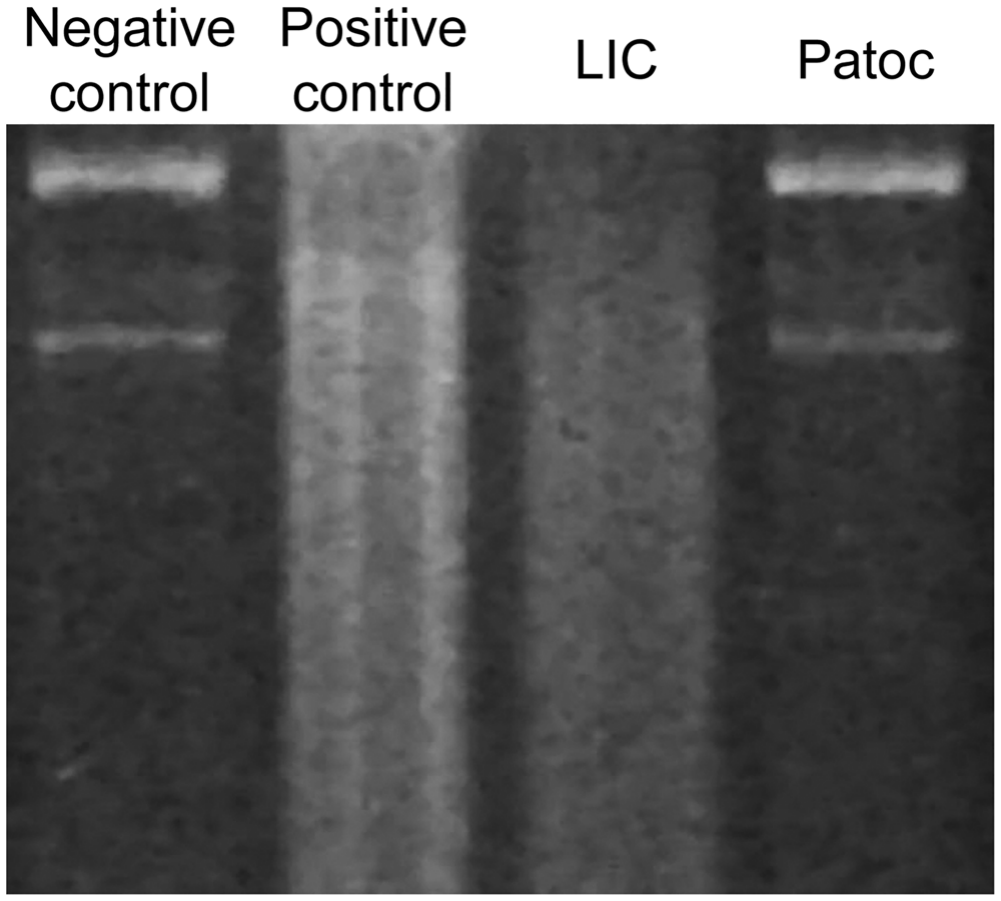
Pathogenic but not saprophyte *Leptospira* spp. degrade DNA. Representative analysis of DNA digestion by gel electrophoresis. From left to right: plasmid DNA (100 ng/μL) after incubation with PBS (negative control), DNase I (positive control), and live *Leptospira interrogans* serovar Copenhageni (LIC) or *Leptospira biflexa* serovar Patoc (Patoc) (1x10^8^/mL) after 60 minutes of incubation at 37°C.

According to our results, some *Leptospira* were able to evade NETs. Because some microorganisms evade NETs due to nuclease activity, we then studied whether *Leptospira* exerts this enzyme activity by incubating plasmid DNA with pathogenic and saprophyte *Leptospira*, or with exogenous DNase as a positive control. Remarkably, while the treatment of DNA with either DNase or the pathogenic LIC resulted in marked DNA degradation, the saprophyte Patoc had no effect (Fig 5).

### Role of NETs in a murine model of experimental leptospirosis

After having characterized the formation of NETs induced by *Leptospira* spp. by human neutrophils using an *ex vivo* model, to further characterize the role of NETs in leptospirosis, we extended our studies to an *in vivo* model. For this purpose, we used a recently characterized murine model based on C57BL/6J mice infected with pathogenic LIC [19,27] after depletion or not of neutrophils by previous treatment with the mAb1A8 [28].

**Fig 6:**
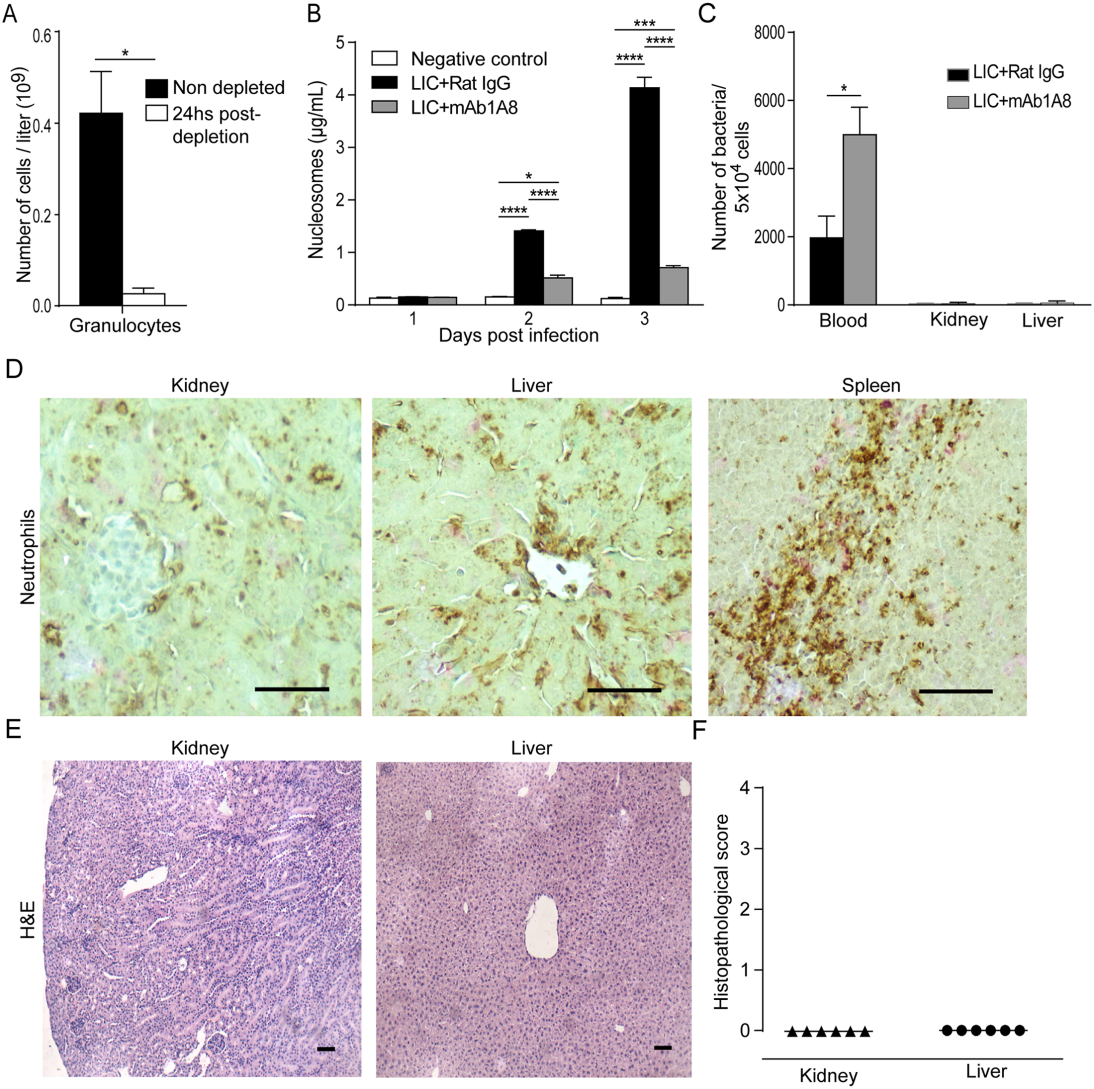
Early role of NETs in murine experimental model. **(A)** Granulocytes were quantified in blood samples taken from C57BL/6J mice treated or not with mAb1A8 antibody with a veterinarian hematology counter. Bars represent the mean ± SEM. * p <0.05. **(B)** C57BL/6J male weanlings were inoculated intraperitoneally with 200 μL of 1x10^7^/mL pathogenic *Leptospira interrogans* serovar Copenhageni (LIC) and 0.25 mg of non-immune rat IgG or purified anti-Ly6G rat mAb1A8 every 48 h. Blood was collected by retro-orbital venous puncture and circulating nucleosomes were measured by ELISA *p <0.05, ***p <0.001, ****p <0.0001. **(C)** Blood, kidney, and liver samples of non-depleted and mAb1A8-depleted LIC-infected mice were collected at 3 days post-infection. Leptospiral DNA was quantified by real-time PCR and normalized to host cell number. Bars represent standard error of the mean (SEM) of assays from two independent assays; *p <0.05. **(D)** Kidney, and liver tissues samples of non-depleted animals were immunostained for neutrophils using anti-Ly6G rat mAb1A8 at 3 days post-infection. Representative hematoxylin and eosin (H&E) stained kidney and liver tissues samples of non-depleted animals at 3 days post-infection showing histology **(E)** and inflammation score **(F)** do not showed any alteration after analysis (n = 6–10 mice). Scale bar indicates 50 μm.

After depletion of more than 90% of neutrophils (Fig 6A), we analyzed the formation of NETs in blood from mice inoculated with LIC during the first 3 days post-infection (dpi), by measuring the levels of nucleosomes in the plasma of infected animals. We found that infection with LIC triggered the generation of intravascular NETs as early as 2 dpi (Fig 6B) and that levels of circulating NETs induced by LIC were significantly reduced in neutrophil-depleted mice (Fig 6B) suggesting that neutrophils are the main source and that perhaps other cells also contribute to extracellular traps since as well as neutrophils, monocytes [29], eosinophils [30], basophils [31], and even mastocytes [32] also produce extracellular traps.

### Neutrophil depletion increases bacteremia

To obtain a deeper insight into the role of NETs in the murine model of leptospirosis, we next evaluated the bacterial burden in blood, kidney and liver in animals depleted or not of neutrophils at 3 dpi. In concordance with the decreased levels of nucleosomes, animals depleted of neutrophils had significantly higher bacteremia than those that were not depleted; however, the bacterial burden in kidney and liver was several orders lower and did not show differences (Fig 6C). We also evaluated the presence of neutrophils in these organs. Interestingly, immunohistochemistry studies revealed a low number of neutrophils in the infected organs in non-depleted animals (Fig 6D). In addition, histopathological analysis of non-depleted mice showed an absence of significant cell exudate or necrosis (Fig 6E and 6F). Taken together, our results suggest that in the leptospiremic phase of infection, murine neutrophils have a major role in preventing *Leptospira interrogans* dissemination through induction of the intravascular formation of NETs.

### Early heightened bacteremia increases late kidney bacterial burden

**Fig 7:**
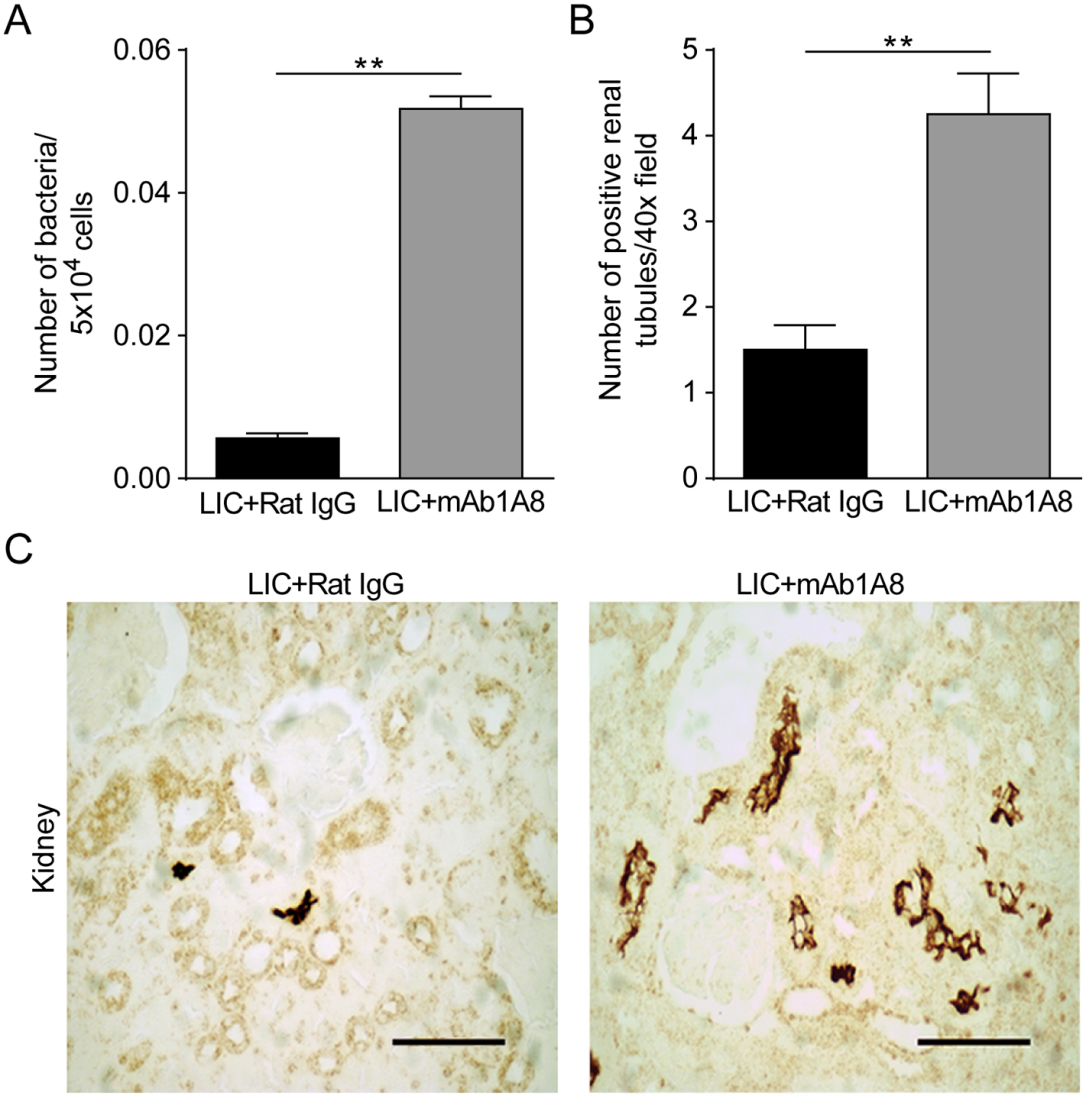
Renal bacterial burden at the beginning of the leptospiruric phase and immunohistochemistry analysis. **(A)** C57BL/6J male weanlings received 0.25 mg of non-immune rat IgG or purified anti-Ly6G rat mAb1A8 every 48 h and 200 μL of 1x10^7^/mL pathogenic *Leptospira interrogans* serovar Copenhageni (LIC). Kidney samples of non-depleted and neutrophil-depleted LIC-infected mice were collected at 14 days post-infection. Leptospiral DNA was quantified by real-time PCR and normalized to host cell number. Bars represent mean ± SEM of two independent experiments; **p <0.01. **(B)** Positive renal tubules were counted under a 40x field. Bars represent mean ± SEM of three independent observations; **p <0.01. **(C)** Representative positive slides of immunohistochemical staining of LipL32 in kidney from LIC-infected mice rat IgG-treated or mAb1A8-treated. The results are representative of two different experiments (n = 6–10 mice). Slides were counterstained with hematoxylin. Scale bar indicates 50 μm.

**Fig 8:**
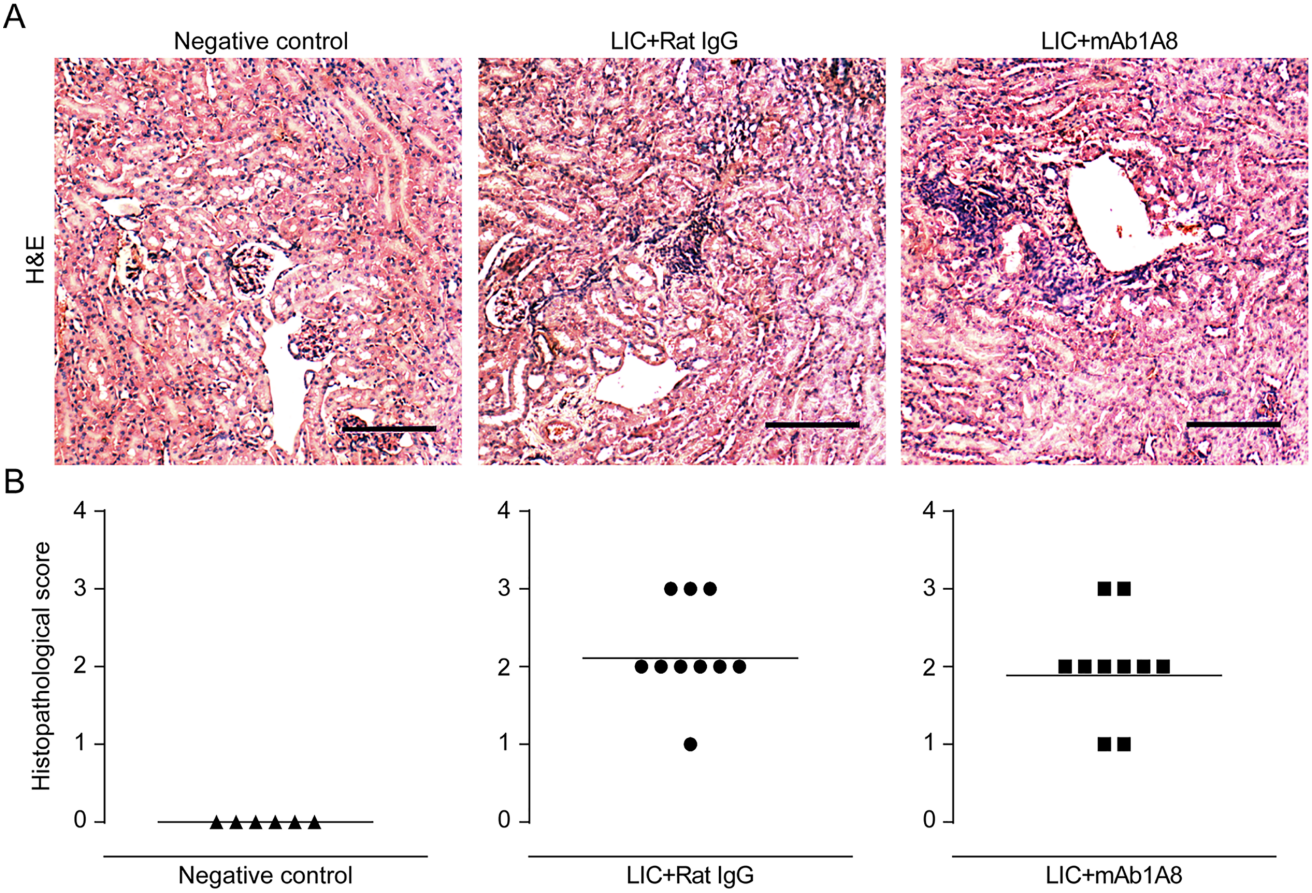
Renal histopathology at the beginning of the leptospiruric phase. C57BL/6J male weanlings received 0.25 mg of non-immune rat IgG or purified anti-Ly6G rat mAb1A8 every 48 h and 200 μL 1x10^7^/mL pathogenic *Leptospira interrogans* serovar Copenhageni (LIC). Representative hematoxylin and eosin (H&E) stained kidney sections from 14 days post-infection showing histology **(A)** and inflammation score **(B)** from uninfected mAb1A8-treated mice, rat IgG-treated LIC-infected mice, and mAb1A8-treated LIC-infected mice (n = 6–10 mice). Scale bar indicates 50 μm.

It has been shown that the level of early bacteremia is linked to a later kidney bacterial burden and tissue damage [33]. Having seen that the depletion of neutrophils resulted in less intravascular NETs and higher bacteremia during the acute host response, we studied whether the increased number of circulating bacteria triggered a higher kidney bacterial burden and eventually more inflammation at a later point after infection. For this aim, the kidneys of mice depleted or not of neutrophils were studied at 14 dpi for bacterial burden and histopathology. Results showed higher bacterial burden, detected either by qPCR or immunohistochemistry, in the kidneys of LIC-infected mice depleted of neutrophils compared to non-depleted mice (Fig 7A–7C). The histopathological analysis revealed an interstitial nephritis of mild to moderate intensity in nearly all infected animals with no differences between non-depleted and neutrophil-depleted mice (Fig 8A and 8B). Collectively, these data show that intravascular NET formation not only promotes bacteria snare and prevent extensively dissemination, but also protects the host from additional kidney bacterial burden.

## Discussion

Our results demonstrated that *Leptospira* spp. were able to induce DNA extracellular traps from human neutrophils and suggest that NETs are critical for the prevention of early bacteria dissemination in a murine model of leptospirosis.

The observation that the saprophyte Patoc was less effective in triggering NETosis in human neutrophils than the pathogenic LIC indicated that pathogenicity is a relevant factor for NET formation mediated by *Leptospira* spp. In addition, neutrophil stimulation with live bacteria also resulted in higher NET formation than dead bacteria, showing that bacteria viability is also required for an efficient NETosis or a proper recognition by neutrophils. A major characteristic of *Leptospira* spp. compared to other bacteria is their great and particular motility. Since it has been recently shown that neutrophils can sense microbial size [34], it is conceivable that they might also sense the particular motility of *Leptospira* spp. However, the amount of DNA released by neutrophils incubated with two immotile mutants was similar to the wild-type strain, indicating that motility has no significant influence on NET formation.

Although NETs trap and kill microbes [11,12,35], some bacteria evade entrapment in the NET due to the release of different virulence factors including nucleases [23]. We found that NETs dramatically reduced *Leptospira interrogans* survival from over 80% to less than 5% in 180 minutes. Furthermore, the presence of a recombinant DNase restored only 30% of viability, suggesting either that the killing process is very fast and/or that even in the absence of the DNA web, which can be bactericidal *per se* [36], the microbicidal proteins still exert their effects. In this regard, we showed that LIC was susceptible to the cytotoxic activity of recombinant histone H4. In addition, we also observed that pathogenic LIC and LIM, but not saprophyte Patoc, have the ability to degrade DNA. Although these data suggest that pathogenic species appear to exert nuclease activity, because we have only studied two pathogenic strains and only one saprophyte, our statement needs to be further confirmed by studying other species and strains. A membrane-bound protein has been described and associated with NETs escape for *Streptococcus pneumoniae* [37], *Streptococcus sanguinis* [38], and group A *Streptococcus* [39]. An even more sophisticated mechanism has been described in *Staphylococcus aureus* related to the secretion of a nuclease and an adenosine synthase that convert NETs to deoxyadenosine, which triggers the caspase-3-mediated death of macrophages [40]. Whether such a mechanism also operates in *Leptospira* spp. remains to be investigated.

Interestingly, although first full genome analysis did not include any annotation for nucleases [41], in more recent years, several nucleases or potential nucleases have been annotated in partially sequence *Leptospira* spp. genome, usually as hypothetical proteins, as it was found in NCBI databases. In any case, it suggests that the ability of *Leptospira* spp. to degrade DNA of extracellular traps is a potential advantage, and is probably a pathogenic determinant because it allows a percentage of bacteria to escape from the NETs entrapment.

Although it has been considered that guinea pigs and hamsters were the appropriate host for *in vivo* studies [42], it has been recently demonstrated that, although less susceptible, the mouse can also be used for pathogenesis studies [19,27,33]. The fact that plasmatic levels of circulating nucleosomes dramatically increased after challenge with LIC indicated that NETosis is a mechanism of host defense in early leptospirosis. However, the fact that LIC disseminates to different organs indicates that some bacteria evade NETs attack. Our observation of a nuclease activity in LIC could certainly provide the bacteria with an escape strategy.

If these data were confirmed in human plasma from leptospirosis-infected patients, nucleosomes could be a potential biomarker with which to detect active leptospirosis. Additionally, it would be important to elucidate the occurrence of NETosis in infected patients from leptospirosis endemic areas, such as Iquitos, Peru [43], as persistent NETosis has been related to kidney injury [44].

It has been described that as well as neutrophils, monocytes [29], eosinophils [30], basophils [31], and even mastocytes [32] also produce extracellular traps. Our observation that neutrophil depletion with mAb1A8 treatment reduced almost 80% the plasmatic levels of circulating nucleosomes suggest that neutrophils are the main source of circulating extracellular traps, but did not discriminate if the remaining neutrophils are the source or other cells also contribute to the release of DNA traps in mice. Of note, neutrophil depletion and reduced NET formation were associated with a higher bacteremia, suggesting that besides other bactericidal properties of neutrophils, NETosis is an important mechanism of the host with which to prevent the increased dissemination of pathogenic *Leptospira* spp. These data are in partial agreement with the reported higher bacteremia of *Enterococcus faecium* in neutrophil-depleted mice [45] although NET formation was not explored in that study.

Considering that bacterial burden has been associated with the inflammation degree of kidney in experimental murine leptospirosis [19,27,33], our finding of nephritis of similar intensity in neutrophil-depleted and non-depleted infected mice at 14 dpi was unexpected. Many factors may be considered in order to explain this situation. First, LIC-induced nephritis was only mild to moderate in C57BL/6J mice, making it more difficult to account for some variations. Second, lympho-monocytic cells constitute the majority of the cell population present in the inflammatory exudate, in agreement with the murine hematological formula, which has a strong preponderance of lymphocytes (75–90%) versus granulocytes (10–25%) [46]. Third, neutrophil-depleted infected mice had higher bacterial burden, which may balance the absence of neutrophils and their eventual contribution to tissue injury by generating reactive oxygen intermediates and releasing lytic enzymes [47,48]; finally, NETs may play a more direct role in the interstitial nephritis induced by LIC.

In conclusion, here, we showed that *Leptospira* spp. are able to induce extracellular traps in human neutrophils. The entrapment in the NETs results in the killing of a significant number of *Leptospira*; however, pathogenic but not saprophytic ones, express nuclease/s that might enable some of them to evade this protective mechanism. Intravascular formation of NETs also occurs *in vivo* upon LIC infection and appears to be a key mechanism by which the host attempts to control bacterial dissemination.

To the best of our knowledge, this is the first time that NETs have been associated with leptospirosis. However, our results need further confirmation in other species, including humans.
